# Immune Checkpoint Inhibitor-Related Hypophysitis and Pituitary Dysfunction: A Systematic Review of Diagnosis and Management

**DOI:** 10.7759/cureus.98717

**Published:** 2025-12-08

**Authors:** Pooja SirDeshpande, Soorya Bavikeri Shivakumara Hegde, Hassan Akhtar

**Affiliations:** 1 Endocrinology, Diabetes and Metabolism, London Northwest University Healthcare NHS Trust, London, GBR; 2 Acute and General Internal Medicine, University Hospitals of North Midlands NHS Trust, Stafford, GBR; 3 Acute Medicine and Endocrinology, University Hospitals of North Midlands NHS Trust, Stafford, GBR

**Keywords:** atezolizumab, cemiplimab, ctla-4 inhibitors, immune checkpoint inhibitor, ipilimumab, nivolumab, pd-1 inhibitors, pd-l1 inhibitors, pembrolizumab, tremelimumab

## Abstract

With an objective to review the clinical presentation, diagnosis, and management of immune checkpoint inhibitor (ICPi)-associated hypophysitis and pituitary dysfunction, we conducted a systematic review of 84 studies (2005-2025) involving 7,259 patients evaluated pituitary immune-related adverse events (irAEs) linked to CTLA-4 inhibitors, programmed cell death protein 1 (PD-1)/programmed cell death-ligand 1 (PD-L1) inhibitors, and combination therapies. Data included ICPi type, demographics, cancer type, treatment duration, imaging, pituitary dysfunction, symptoms and management.

Following statistical analysis, the weighted mean of male patients was 68.3%, with a pooled mean age of 63.9 years. Common symptoms included fatigue, headache, hyponatraemia, nausea, anorexia, and neuropsychiatric changes. While MRI is a key diagnostic tool, it may not always detect subtle or early-stage pituitary involvement.

In the CTLA-4 group, patients received ipilimumab for 2-12 cycles (mean: 3.3) before hypophysitis onset. In the PD-1/PD-L1 group, median time to onset was 28 weeks (range: 10-46 weeks). Hypophysitis induced by CTLA-4 inhibitors, particularly ipilimumab and CTLA-4-based combination therapies, were more commonly associated with hypopituitarism. In contrast, isolated adrenocorticotropic hormone (ACTH) deficiency, often linked to PD-1/PD-L1 inhibitors, presents as secondary adrenal insufficiency without distinct MRI abnormalities.

Reported MRI abnormalities included hypophysitis, pituitary stalk abnormalities, pituitary enlargement, microadenoma, pituitary atrophy, and empty sella. The most common biochemical abnormalities in the combination group were hypopituitarism and secondary adrenal insufficiency. High-dose glucocorticoid initiation, careful tapering, and tailored long-term hormone replacement remained the mainstays of management.

In conclusion, our systematic review highlights hypopituitarism as a frequent and often persistent consequence of ICPi-associated hypophysitis. Early recognition through combined clinical, biochemical, and radiological assessment is essential to reduce long-term endocrine morbidity and optimise patient outcomes.

## Introduction and background

Discovered in the late 20th and early 21st centuries, immune checkpoint inhibitors (ICPi) have revolutionised cancer treatment in recent years. Immune checkpoints are small molecules on the surface of immune cells involved in maintaining immune homeostasis [1]. ICPis are the monoclonal antibodies that work by targeting immune checkpoints such as cytotoxic T-lymphocyte-associated protein 4 (CTLA-4), programmed cell death protein 1 (PD-1) and programmed cell death-ligand 1 (PDL-1), thereby enhancing T-cell activation and promoting a robust antitumor immune response. Their introduction has ushered in a new era of immuno-oncology with durable responses across a range of malignancies [2]. ICPi therapy can also trigger autoimmune adverse effects, termed immune-related adverse events (irAEs). Endocrinopathies are among the most common irAEs associated with ICPi therapy and include hypophysitis, thyroid dysfunction (thyroiditis, post-immune checkpoint inhibitor hypothyroidism, or Grave’s disease), insulin-deficient diabetes mellitus (DM), adrenal insufficiency (AI), hypoparathyroidism and rarely AVP deficiency, prolactin deficiency or hyperprolactinemia [3]. Hypophysitis, or inflammation of the pituitary gland, is one of the most common ICPi-related endocrinopathies and is mainly associated with anti-CTLA-4 therapy, but can also be encountered with PD1 and PDL-1 or combination therapies. With the growing use of immune checkpoint inhibitor (ICPi) therapy in oncology and the potentially life-threatening consequences of untreated endocrinopathies, it is essential that clinical practitioners are well-informed about the clinical presentation, diagnosis, and management of ICPi-induced endocrine disorders [4,5]. The aim of our study was to perform a systematic review of ICPi-associated pituitary dysfunction, including its clinical presentation, diagnosis, and management.

## Review

**Figure 1:**
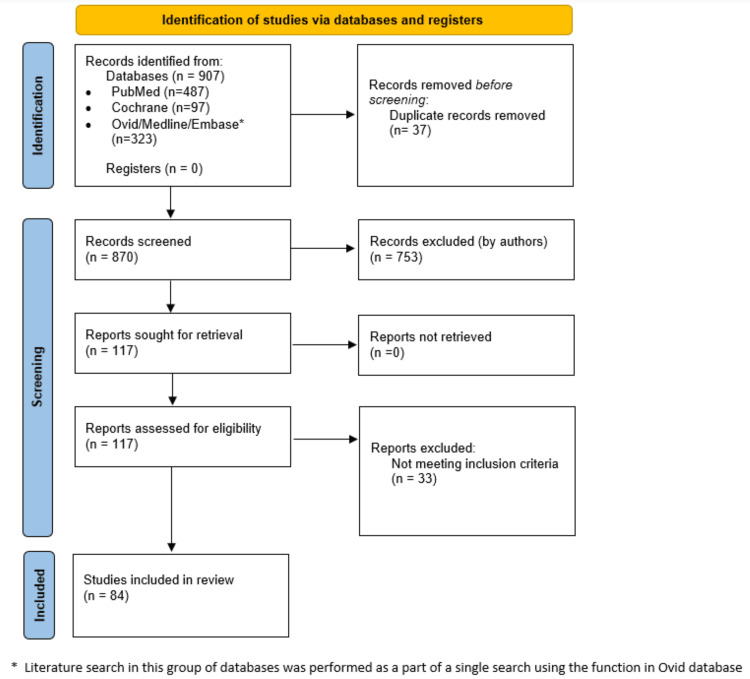
Summary of literature search and selection

This review was conducted in accordance with the Preferred Reporting Items for Systematic Reviews and Meta-Analyses (PRISMA) reporting guidelines (Figure 1). Literature search was carried out using the advanced search option in PubMed, Cochrane, Ovid, Medline and Embase from 2005 to 2025. Original articles that have published results were identified by a literature search and by examining the references of published articles by two authors (PS and SH). The following keywords or corresponding Medical Subject Headings (MeSH) terms were used: “Immune checkpoint inhibitor,” “CTLA-4,” “Ipilimumab,” “Tremelimumab,” “PD-1,” “Nivolumab,” “Pembrolizumab,” “Cemiplimab,” “PD-L1,” “Atezolizumab,” “Durvalumab,” “Avelumab,” “Hypophysitis,” “Hypopituitarism,” “Pituitary dysfunction.”

The search focused on original studies investigating pituitary dysfunction induced by ICPi monotherapy or combination therapy. Our inclusion criteria encompassed all prospective or retrospective single- or multicentre randomised case-control studies, cohort studies, observational audits or quality improvement studies, case series with literature reviews, and meta-analyses. Only articles published in English with available results were included. We excluded single case reports, abstracts without full-text published articles, expert opinions, editorials, and narrative reviews.

Individual case reports were included for the PD-L1 inhibitor category due to the limited availability of higher-quality evidence within this class. These reports were presented separately in the review and were not included in the statistical analysis. In cases of duplicate publications, ambiguity, or multiple reports on the same study population, only the most recent, relevant, or comprehensive publication was included in the analysis. Any discrepancies in study selection were resolved by consensus.

A total of 907 articles were identified through the literature search, of which 117 met the eligibility criteria. Ultimately, 84 articles were included in the final review (Figure 1).

Data were extracted under the following headings: type of immune checkpoint inhibitor (ICPi), name of the drug, dose of ICPi, duration or number of cycles administered, total number of patients, number of patients analysed, sex distribution (number of males/females), type of cancer, imaging modality used, imaging results, type of pituitary dysfunction or immune-related adverse events (irAEs), clinical symptoms, and treatment administered (Tables 1-3) Individual case reports within the PD-L1 category were excluded from the final statistical analysis and instead summarised in a separate table (Table 4).

**Table 1:** Literature included and analysed for CTLA-4 monotherapy category Literature included and analysed for the CTLA-4 monotherapy category HRT: hormone replacement therapy; IIH: idiopathic intracranial hypertension; ICPi: immune checkpoint inhibitor; NR: not reported

Author (Year of Publication)	Type of ICPi	Name of the Drug	Dose of ICPi	Duration of ICPi	Total Number of Analysed Patients	Age (Years)	Gender	Type of Cancer	Results of Imaging	Type of Pituitary Dysfunction	Symptomatology	Treatment Given
Blansfield et al. (2005) [6]	CTLA-4	Ipilimumab	3-9 mg/kg	5-9 doses	8	31-61	100% Male	Melanoma, RCC	IIH - 8/163 (4.5%)	Hypopituitarism	Fatigue, headache, diarrhoea, insomnia, constipation	Intravenous dexamethasone (4 mg every 6 hours) for 7 days and then tapered to maintenance doses of hydrocortisone with HRT
Yang et al. (2007) [7]	CTLA-4	Ipilimumab	3 mg/kg stat f/b 1 mg/kg in cohort A 3 mg/kg in cohort B	Every 3 weeks - total of 4 doses	61	31-70	45/61 (73.7%) Male	RCC	IIH - 2/61 (3.27%)	Hypopituitarism	Diarrhoea, rash, arthritis	Systemic dexamethasone and steroid enemas for enteritis, Hydrocortisone for hypophysitis
Dillard et al. (2010) [8]	CTLA-4	Ipilimumab	10 mg/kg in case 1. No mention of case 2	4 doses in case 1. No mention in case 2	2	50, 67	Both males	Prostate cancer	IIH	Hypopituitarism	Headache with eye pain, asthenia, anorexia, labile moods, insomnia, fever and chills	Case 1 - Prednisone 60 mg daily and tapered to 10 mg/day. Case 2 - Prednisone 120 mg/day
Lammert et al. (2013) [9]	CTLA-4	Ipilimumab	3 mg/kg in 6 and 10 mg/kg in 1 patient	1-4 cycles	7	31-77	6/7 (85.71%) Male	Melanoma and prostate cancer	IIH	Hypopituitarism	Headache, fatigue, dizziness	Corticosteroids + HRT
Chodakiewitz et al. (2014) [10]	CTLA-4	Ipilimumab	3 mg/kg	4 cycles	3	45-65	3 females	Melanoma	Hypophysitis in all 3 patients	Hypopituitarism	Headache, fatigue	Glucocorticoid and HRT
Iwama et al. (2014) [11]	CTLA-4	Ipilimumab	3 mg/kg in 4 patients, 10 mg/kg in 16 patients	3 - 12 cycles	20	34-78	15/20 (75%) Male	Melanoma, prostate cancer	Hypophysitis in 4/20 (20%) patients	Hypopituitarism	Fatigue, headache, vision changes and weakness	NR
Faje et al. (2014) [12]	CTLA-4	Ipilimumab	3 mg/kg (13 patients) 10 mg/kg (4 patients)	Every 3 weeks - total of 4 doses	17	68.2 +/- 2.4	15/17 (88.2%) Male	Melanoma	IIH in 17/154 (11%)	Hypopituitarism	Headache, fatigue, nausea, reduced appetite, cold intolerance, hot flashes, dizziness, confusion, blurry vision	High-dose glucocorticoids (prednisone 60 mg daily) followed by physiological replacement dose
Araujo et al. (2015) [13]	CTLA-4	Ipilimumab	3 mg/kg in 49.1% and >3 mg/kg in 50.9%	>3 vs<3	71	Median 60 (31-82)	56/71 (78.87%) Male	Melanoma	Hypophysitis in 44/57 (77.1 %)	Hypopituitarism	Fatigue, headache, arthritis, low libido	Glucocorticoids in 46/54 (85.2 %) and HRT in 62/66 (93.9 %)
Lam et al. (2015) [14]	CTLA-4	Ipilimumab	3 mg/kg × 3 doses in nine 10 mg/kg × 4 doses in one	4 cycles in 8 patients, 2 cycles in 2 patients	10	46-70	9/10 (90%) Male	Melanoma	Hypophysitis in 5 patients (50%)	Hypopituitarism	Fatigue, nausea, weakness, hyponatraemia	High-dose glucocorticoids - 6 patients with visual compromise (Physiological doses in 4 patients)
Albarel et al. (2015) [15]	CTLA-4	Ipilimumab	3-10 mg/kg	2-4 cycles	15	55.5+/-11.2	10/15 (66%) Male	Melanoma	Hypophysitis in 15 patients (11.45%)	Hypopituitarism	Headache, asthenia, weakness, dizziness, reduced libido	Glucocorticoid therapy
Le Min et al. (2015) [16]	CTLA-4	Ipilimumab	3 mg/kg in 17 patients, 10 mg/kg in 25 patients	Every 3 weeks - total of 4 doses	25	NR	19/25 (76%) Male	Melanoma	IIH 16.1% (19/118) in males, 8.7% (6/69) in females, NET - 13.3% (25/187)	Hypopituitarism	Headache and fatigue	High-dose glucocorticoid (30 mg hydrocortisone) in 15 patients
Faje (2016) [17]	CTLA-4	Ipilimumab	3 or 10 mg/kg	NR	57	Mean 68.2	44/57 (77.19%) Male	Melanoma	IIH - in 44 patients	Hypopituitarism	Headache and fatigue	Physiologic or moderately supraphysiologic doses of glucocorticoids
Mahzari et al. (2015) [18]	CTLA-4	Ipilimumab	3 mg/kg in 5 patients, 10 mg/kg in 1 patient	NR	6	54-80 years	4 males and 2 females	Melanoma	IIH	Hypopituitarism	Headache	High-dose prednisone in case 1
Brilli et al. (2017) [19]	CTLA-4	Ipilimumab	3 mg/kg or 10 mg/kg	Every 3 weeks for a total of four doses	273	64.13 ± 9.6 years	165/273 (60.44%) male	Melanoma, prostate cancer	Hypophysitis in 9/273 patients (3.3%)	Hypopituitarism	Headache, fatigue, general discomfort	Physiological replacement therapy (cortisone acetate 37.5 mg/day)
De Sousa et al. (2018) [20]	CTLA-4	Ipilimumab	3 mg/kg	Every 3 weeks - total of 4 doses	46	63+/-4.2	6/9 Male (66%)	Melanoma	IIH - 9/46 (19.56%)	Hypopituitarism	Headache, fatigue, anorexia, light-headedness, low mood, weight loss	Supraphysiological glucocorticoid therapy in 7 patients and replacement doses in 2 patients
Faje et al. (2018) [21]	CTLA-4	Ipilimumab	1, 3 or 10 mg/kg	No. of treatment cycles at time of diagnosis 3.3 +/- 0.1 Total no. of treatment cycles 4.4+/-0.5	98	63.4 +/- 1.3	67/98 (68.36%) Male	melanoma	IIH	Hypopituitarism	Headache, Fatigue, nausea/appetite loss, dizziness, altered mental status, myalgias/arthralgias	Median glucocorticoid doses were 5.5 mg (IR - interquartile range, 3.75-7.5 mg) and 22.4 mg (IR, 11.0-35.7 mg) in the low-dose and high-dose groups
Snyders et al. (2019) [22]	CTLA-4	Ipilimumab	(13/15) had 3 mg/kg (2/15) had 10 mg/kg	3.0 cycles (95% CI 2.6 to 3.4) vs. 3.5 cycles (95% CI 3.3 to 3.7), P=0.087	15	Median of 62.1 yrs	10/15 (66.7%) Male	Melanoma	15/117 (12.8%) developed IIH	Hypopituitarism	headaches, fatigue, hyponatraemia, blurry vision, weakness, and decreased libido	High-dose corticosteroids were administered to 12 of the patients (≥40 mg prednisone daily). Low-dose corticosteroids in 3 patients (<80 mg prednisone daily)
Atkins and Ur (2020) [23]	CTLA-4	Ipilimumab	NR	NR	2	59 and 69 years	1 male, 1 female	1 RCC and 1 melanoma	Normal in one and enlarged stalk with loss of bright spot in the other	Adrenal insufficiency Panhypopituitarism	Headache, fatigue, loss of libido, erectile dysfunction, diplopia, nausea, hypotension	69/F: Only HRT 59/F: discontinuation + supraphysiological doses of steroids + HRT

**Table 2:** Literature included and analysed for PD-1/PD-L1 category Literature included and analysed for the PD-1 / PD-L1 category ICPi: immune checkpoint inhibitors; HCC: hepatocellular carcinoma; RCC: renal cell carcinoma; ACTH: adrenocorticotropic hormone; NSCLC: non-small cell lung cancer; NR: not reported; ICI-HP: immune checkpoint inhibitor-induced hypopituitarism

Author (Year)	Type of ICPi	Name of the Drug	Dose of ICPi	Duration of ICPi	Total Analysed Patients	Age (years)	Gender	Type of Cancer	Results of Imaging	Type of Pituitary Dysfunction	Symptomatology	Treatment Given
Baxi et al. (2018) [24]	PD1, PDL-1	Nivolumab, Pembrolizumab, Atezolizumab	NR	Median of nine courses (Range: 3–21)	13	NR	NR	NSCLC, Melanoma, RCC, bladder cancer of head and neck, SCC	NR	Hypopituitarism	Fatigue, arthralgia	NR
Faje et al (2019) [25]	PD1	Nivolumab, Pembrolizumab	NR	NR	22	NR	NR	Melanoma, Lung cancer, Ovarian cancer, Colorectal, Breast and squamous cell cancer of the oropharynx	Hypophysitis	Hypopituitarism	Fatigue, nausea, loss of appetite, myalgias/arthralgias, headaches, dizziness, and altered mental status	Physiologic glucocorticoid doses or mildly supraphysiologic doses
Lupi et al. (2019) [26]	PD1, PDL1	Nivolumab, Pembrolizumab, Atezolizumab	Case 1: Atezolizumab: 1200mg/3 weeks, Case 2: Nivolumab: 3mg/kg every 2 weeks, Case 3: Pembrolizumab: 2mg/kg every 3 weeks, Case 4: Nivolumab: 3mg/kg every 2 weeks	Case 1: After the fourth dose, Case 2: After the sixth cycle, Cases 3 and 4: 4 months	4	78,80,43, 60	3 males and 1 female	2 metastatic melanoma, 2 NSCLC	Did not reveal specific signs of pituitary inflammation	Hypopituitarism: central hypoadrenalism and hyponatraemia	Nausea, headache, vomiting, drowsiness, fatigue, muscle weakness, fever, asthenia	Hydrocortisone
Levy et al. (2020) [27]	PD1, PDL-1	Nivolumab, Pembrolizumab, Tislelizumab, Atezolizumab	Pembrolizumab every three weeks at a dose of 2 mg/kg for 1 patient with melanoma. NR for other 16 patients	Median of nine courses (Range: 3–21)	17	64 ± 8.2 years	13/17(76.5%) Male	Melanoma, NSCLC, HCC, RCC, cutaneous T cell lymphoma	No pituitary abnormality	Adrenocorticotropic hormone (ACTH) deficiency; other pituitary deficiencies were less common	Fatigue, nausea or loss of appetite, headache	High-dose hydrocortisone followed by oral hydrocortisone at replacement doses (15 to 20 mg per day)
Bellastella et al. (2021) [28]	PD1, PDL-1	Nivolumab, Pembrolizumab, Avelumab, Atezolizumab, Durvalumab	NR	NR	37 PD1 and 17 PDL1	65 ± 10.7 Longitudinal study- 70.6 ± 10.4	35/54 (64.81%) Male Longitudinal study: 10/13(76.92%) Male	Lung cancer, Melanoma	NR	Hypopituitarism: Low ACTH and IGF-1, raised prolactin	NR	NR
Han et al. (2022) [29]	PD1	Camrelizumab	200 mg every 2 weeks with oral Apatinib 250 mg daily	11 cycles of Camrelizumab in 1st case 10 cycles of Camrelizumab in 2nd case 8 cycles of Camrelizumab in 3rd case	3	60,68,69 years	All 3 males	NSCLC, HCC, RCC	Hypophysitis, empty Sella	Hypopituitarism	General malaise and appetite loss, syncope, hypotension, diarrhoea	Methylprednisolone (2 mg/kg) for maximum of 5 days, followed by maintenance dose of hydrocortisone/prednisolone
Chen et al. (2023) [30]	PD-1, PDL-1	Sintilimab, Pembrolizumab, Toripalimab, Camrelizumab, Tislelizumab, Nivolumab, Atezolizumab	Sintilimab (200 mg, 3 weekly), Pembrolizumab (200 mg, 3 weekly), Toripalimab (240 mg, 3 weekly), Camrelizumab (200 mg, 2 weekly), Tislelizumab (240 mg, 2 weekly), Nivolumab (unknown, 2 weekly), Atezolizumab (1200 mg, 3 weekly), drug A/B in clinical trial (unknown, 3 weekly)	NR	28	61.2±10.9	16/28 (57.14%) Male	Cervical, Nasopharyngeal, Lung, Oesophageal, Gastric HCC, cholangiocarcinoma, RCC, Melanoma, Breast cancer	Hypophysitis	Isolated ACTH deficiency, Primary hypothyroidism, Fulminant type 1 diabetes mellitus	Fatigue, nausea, headache, thirst, polydipsia	Intravenous corticosteroids, with a median dose of 200 (50–250) mg
Yang et al. (2024) [31]	PD1	Nivolumab, Pembrolizumab, Sintilimab, Camrelizumab, Tislelizumab	NR	Average duration of the use of PD‐1 inhibitors: 13.50 weeks (range: 3.00–56.00 weeks)	14	65.00 ± 6.82 (Age range: 51.00–75.00 yrs old)	11/14 (78.57%) Male	Malignant melanoma, Oesophageal, Lung ca, Colorectal, HCC, Bladder	No abnormalities on MRI	Isolated ACTH deficiency	Fatigue, loss of appetite, nausea, vomiting, fever, memory loss, disturbance of consciousness, visual impairment, headache	Glucocorticoid replacement therapy (Prednisolone 2.5 to 7.5 mg/day or equivalent)
Suzuki et al. (2024) [32]	PD-1	Nivolumab, Pembrolizumab	NR	NR	194	<75 =156 (80.4%) ≥75 = 38 (19.6%)	146/194(75.3%) Male	NSCLC	NR	Pituitary dysfunction, thyroiditis	NR	None of the patients with pituitary dysfunction was treated with high-dose glucocorticoids
Iwamoto et al. (2024) [33]	PD1, PDL-1	Nivolumab, Pembrolizumab, Atezolizumab, Avelumab	NR	Time from ICI administration to diagnosis of ICI-HP was 125 (56-212) days	13	66 (57-69)	10/13 (76.92%) Male	Melanoma, RCC, HCC, GI, NSCLC, Gingival, unknown primary	Empty Sella, thickening of pituitary stalk, hypophysitis, normal pituitary	Isolated ACTH deficiency	General malaise, decreased appetite, headache, hypoglycaemia, decreased consciousness, high fever, amenorrhoea, mood disturbance	NR

**Table 3:** Literature included and analysed for combination therapy category Literature included and analysed for the combination therapy category NR: not reported; AML: acute myeloid leukaemia; HPA: hypothalamic–pituitary–adrenal

Author	Type of ICPi	Name of the drug	Dose of ICPi	Duration of ICPi	Total analysed patients	Age (years)	Gender	Type of cancer	Results of imaging	Type of Pituitary dysfunction	Symptomatology	Treatment given
Miller et al (2016) [34]	CTLA-4, CTLA-4+PD-1	Ipilimumab, Ipilimumab+Nivolumab	NR	3-4 doses	5	52-72	3/5 (60%) male	Melanoma, papillary thyroid carcinoma and RCC	IIH	Hypopituitarism	severe fatigue, confusion, hypotension, headache, hyponatremia	Stress-dose glucocorticoids (eg, hydrocortisone 100 mg)
Caturegli et al (2016) [35]	CTLA4, CTLA4+PD1	Tremelimumab, Ipilimumab + Pembrolizumab, Ipilimumab + Nivolumab	Tremelimumab 10mg/kg every 4 weeks, Ipilimumab 3mg/kg every 3 weeks No mention for the rest	Case 3: 3 doses of Tremelimumab, 1 dose of Ipilimumab Case 4- 4 doses of ipilimumab Case 5 - 4 doses	6	79, 30,59, 65,67,58	2/5(40%) Males	Pleural mesothelioma, melanoma for combi therapy	IIH	Hypopituitarism	Diarrhoea, profound fatigue and vomiting	Glucocorticoids (hydrocortisone, 100 mg four times per day; and methylprednisolone, 250 mg/day)
Ariyasu et al (2017) [36]	PD1, PD1+CTLA4	NR	NR	NR	5	Mean age of 65.4 years (range=58-72 years)	All 5 male	NSCLC and SCLC	Hypophysitis (in 1/5)	Hypopituitarism (isolated ACTH deficiency)	Fatigue and anorexia	NR
Malikova et al (2018) [37]	CTLA-4, PD-1	Ipilimumab, Pembrolizumab	Ipilimumab 3 or 10mg/kg Pembrolizumab 200mg/dose	Ipilimumab: 4 cycles in patient 1 NR in the rest	28	58±13 years	9/28 (32.1%) Male	Melanoma	Swollen, enlarged or normal pituitary	Hypopituitarism	Headache, fatigue, fever, malaise, photophobia, anorexia	Corticosteroid therapy at immunosuppression dose
Di Dalmazi et al (2019) [38]	CTLA4, PD1, PDL-1	Ipilimumab, Tremelimumab, Nivolumab, Pembrolizumab, Cemiplimab, Atezolizumab Avelumab, Durvalumab	NR	NR	273	Older ages (61 ± 11.7)	199/273(72%) male	Skin, Lung, Genitourinary	IIH (MRI normal in 36% patients)	Hypopituitarism	Headache, visual changes and hormonal disturbances	High-dose glucocorticoids to be considered to patients with compressive symptoms
Guerrero et al (2019) [39]	CTLA-4, CTLA-4+PD-1, PD-1	Ipilimumab, Ipilimumab+Nivolumab, Nivolumab	NR	3 doses (median 3, range 1-69)	689	63(20-88)	409/633 (64.6%) Male	Melanoma, lung and renal	IIH	Hypopituitarism	Headache, fatigue, visual defects	High dose steroids may be considered for patients with compressive symptoms + HRT
Garon-Czmil et al (2019) [40]	CTLA-4, PD-1, CTLA-4+PD-1	Ipilimumab, Nivolumab, Pembrolizumab, Ipilimumab+Nivolumab	Ipilimumab: 235(15-330)mg per course Nivolumab: 242(123-354)mg per course Pembrolizumab: 136(88-190)mg per course Ipilimumab+Nivolumab: 74(60-90) +229(190-260)mg per course	Ipilimumab: 4(1-7) courses, Nivolumab: 10(1-33) courses Pembrolizumab: 7(1-19) courses Ipilimumab+Nivolumab: 3(2-4) courses	94	64·5 years (+/−14·1)	45/94 (47.87%) Male	Melanoma, RCC, Colon, Pleural, NSCLC, SCLC	IIH - Empty Sella turcica or normal pituitary imaging	Hypopituitarism	Fatigue and cephalalgia	Hydrocortisone supplementation and HRT
Mekki et al (2019) [41]	CTLA-4+PD-1, CTLA-4	Ipilimumab+Nivolumab, Ipilimumab	NR	NR	82	56.9 +/- 12 years, with range of 23-87 years	66 (55.5%) males	Melanoma, NSCLC, RCC, Oesophageal, Breast, Thyroid Rectal, NET	IIH	Hypopituitarism	Headaches	NR
Zhong Wei et al (2019) [42]	CTLA-4, PD-1	Ipilimumab, Pembrolizumab	3mg/kg in 5 patients 2mg/kg in 1 patient	3,3,4,2,3,35 cycles in the 6 patients	6	67 ± 14.3	38/51 (74.5%) Male	Melanoma	IIH (MRI normal in 3)	Hypopituitarism	Headache, nausea, Syncope, tiredness, vomiting, tiredness, rash	Dexamethasone (8-16 mg) in 4 patients. Not given in the other 2
Leiter et al (2020) [43]	CTLA-4, PD-1	Ipilimumab, Pembrolizumab	Case 1: Ipilimumab 10 mg/kg Case 2: pembrolizumab 200 mg/dose	case 1: 8 weeks case 2: 4 cycles	2	75	1/2 (50%) male	urothelial cancer	case 1: Enhancing pituitary nodule case 2: NR	Hypopituitarism	fatigue and headaches.	Prednisolone
Siddiqui et al (2020) [44]	CTLA4 +PD1	Ipilimumab + Nivolumab	3mg/kg	3 cycles (3-4) in 25 patients with hypophysitis	25	Median 65 (32-79)	12/25 48%) Male	Melanoma	Hypophysitis in 25 patients	Hypopituitarism	Headache, fatigue, visual defects (unrelated to hypophysitis)	NR
Bai et al (2020) [45]	CTLA-4, PD-1, PD-L1, CTLA4+PD1, CTLA4+PDL1	Ipilimumab, Nivolumab, Pembrolizumab, Atezolizumab, Durvalumab, Ipilimumab+Nivolumab	Ipilimumab: 3 or 10mg/kg Nivolumab: 1-2 or >/3 mg/kg Pembrolizumab: 2mg/kg	NR	1144	18-44: 7.2% 45-64: 44.99% >65: 47.78%	~64% male	Melanoma, Lung, RCC, Prostate, Gastric, Head/neck, Ovarian	IIH - MRI normal in 93% patients	Hypopituitarism	NR	NR
Kobayashi et al (2020) [46]	CTLA-4, PD-1, PDL-1, CTLA-4+PD-1	Ipilimumab, Nivolumab, Pembrolizumab, Atezolizumab, Ipilimumab+Nivolumab	Ipilimumab 3mg/kg every 3 weeks Nivolumab 2-3mg/kg every 2-3 weeks Pembrolizumab 2mg/kg or 200mg every 3 weeks Atezolizumab 1200mg every 3 weeks	Ipilimumab for 4 cycles NR for the rest	16	67±10 (NSCLC) 69±12 (MM)	79 (73.1%) Male in NSCLC 39 (59.1%) Male in MM	NSCLC and MM	IIH	Hypopituitarism	Fatigue, appetite loss, and weight loss,	Physiological doses of hydrocortisone (10–20mg/day) with HRT
Yano et al (2020) [47]	CTLA-4, PD-1	Ipilimumab, Nivolumab, Pembrolizumab	NR	NR	11	39-70 61.9+/- 9.5 for pituitary irAEs	6/11 (54.54%) Male	MM, NSCLC or gastric	IIH - Thickening of the pituitary stalk, enlargement of the pituitary gland	Hypopituitarism - secondary adrenal sufficiency	NR	Physiological dose of hydrocortisone
Kurokawa et al (2020) [48]	CTLA-4, CTLA4+PD1	Ipilimumab, Ipilimumab+Nivolumab	NR	NR	20	Mean 57.7 (30-86) yrs	10/20 (50%) male	Melanoma	IIH - Enlargement of the pituitary gland and stalk, hypo enhancing lesions	Hypopituitarism	Fatigue, malaise, vomiting, anorexia, numbness, oedema	NR
Seejore et al (2021) [49]	CTLA4 +PD1	Ipilimumab + Nivolumab	Ipilimumab: 3mg/kg 4 weekly Nivolumab: 1mg/kg	1-4 cycles	24	Median 58.8 (23.5-80.9)	125/188 (66%) Male	Melanoma	Hypophysitis in 24 patients (13%)	Hypopituitarism	Fatigue, headache and dizziness	Glucocorticoid therapy in all patients
Kotwal et al (2021) [50]	CTLA4, PD1, CTLA4+PD1	Ipilimumab, Pembrolizumab, Ipilimumab+Nivolumab	NR	NR	26	Mean 61.7 (19.9–93.4)	14/26 (53%) Male	Melanoma and solid organ tumours	IIH in 30/48 (62.5%)	Hypopituitarism, thyroiditis	Headache, diplopia and visual symptoms	High-dose glucocorticoids patients with mass effect symptoms
Kanie et al (2021) [51]	CTLA-4+PD-1, PD-1, PDL-1	NR	NR	NR	20	35 - 87 (Average 66.9)	16/20 (80%) male	NSCLC, RCC, melanoma, stomach, urinary tract, oesophageal, large cell NET, submandibular	Pituitary enlargement, atrophy or no change	Isolated ACTH deficiency	NR	NR
Amereller et al (2021) [52]	CTLA-4, PD-1, CTLA-4+PD-1	Ipilimumab, Ipilimumab+Nivolumab, Pembrolizumab, Nivolumab, Ipilimumab+Pembrolizumab	NR	Median of 4 doses (range 2–14)	56 (60 were primary hypophysitis)	60±14 (22–87) in IIH and 45±16 (15–83) in PH	Male sex - 36/56 (64%) in IIH and 16/60 (27%) in PH	NR	IIH Pituitary enlargement, thickened pituitary stalk or space-occupying intrasellar lesions	Hypopituitarism	Fatigue, headache, nausea, dizziness, visual impairment, Polyuria with polydipsia	NR
Iglesias et al (2021) [53]	CTLA-4, PD-1, PDL-1, CTLA4+PD1	Ipilimumab, Nivolumab, Pembrolizumab, Durvalumab, Ipilimumab+Nivolumab	NR	NR	60	Mean age at diagnosis was 63.2±11.6	37/60 (61.7%) Male	Melanoma, lung, breast, kidney, ovary, gastric, urothelial and colon	IIH	Isolated ACTH deficiency	Fatigue, anorexia, nausea, vomiting and diarrhoea	Hydrocortisone replacement therapy
Iglesias et al (2021) [54]	PD-1, CTLA-4, PDL-1	Nivolumab, Pembrolizumab, Ipilimumab, Durvalumab, Atezolizumab, Tremelimumab, Spartalizumab	NR	NR	37	64.7 ± 8.3 years (Range 46–79)	23/37 (62.2%) Male	Lung, melanoma, Head/neck, Urothelial, RCC, Colon	IIH - abnormal MRI in 3 patients	Hypopituitarism - IAD	Fatigue, malaise, anorexia, nausea, vomiting, syncope	Hydrocortisone (n=31, median 50mg), followed by prednisone (n=5, median 60mg) and methylprednisolone (n=1, median 50mg)
Takagi et al (2021) [55]	CTLA-4+PD-1	Ipilimumab+Nivolumab	NR	NR	22	66+/- 13	17/22 (77%) Male	RCC	NR	Hypopituitarism	Fatigue, weakness, fever	Maintenance dose of steroids
Nguyen et al (2021) [56]	CTLA4, PD1, CTLA4+PD1	Ipilimumab, Tremelimumab, Nivolumab, Pembrolizumab, Ipilimumab+Nivolumab, Pembrolizumab + Ipilimumab	NR	NR	62	Mean 64 (57–67)	48/62(77.4%) male	Melanoma, prostate, RCC, NSCLC, PTC and CMML	IIH in 47/61 (77%)	Hypopituitarism - central adrenal insufficiency	Headache, fatigue, visual defects, polyuria, polydipsia	Glucocorticoid therapy (either physiologic or high dose) at diagnosis
Kobayashi et al (2021) [57]	CTLA-4, PD-1, PDL-1, CTLA-4+PD-1	Ipilimumab, Nivolumab, Pembrolizumab, Atezolizumab, Ipilimumab+Nivolumab	NR	NR	22	NR	NR	Melanoma, NSCLC, RCC	IIH	Hypopituitarism	NR	NR
Takayasu et al (2022) [58]	CTLA-4+PD-1, PD-1, PDL-1	Ipilimumab+Nivolumab, Pembrolizumab, Nivolumab, Durvalumab	NR	NR	17	67.1±9.8	12/17 (70.58%) Male	RCC, Lung, Melanoma	NR	Hypopituitarism - Secondary adrenal insufficiency	General fatigue and general weakness, appetite/weight loss, gastrointestinal symptoms, hypotension, apathy, lethargy, anxiety, fever, arthralgia	NR
Druce et al (2022) [59]	CTLA-4, PD-1, CTLA-4+PD-1	NR	NR	NR	265	Median 65.5	178/265 (67.1%) Male	Melanoma, Genitourinary, head/neck and GI	NR	Central adrenal insufficiency	Fatigue, weakness, poor oral intake, low blood pressure, nausea and vomiting	Glucocorticoid and mineralocorticoid treatment regimens are suggested at physiologic doses
Jessel et al (2022) [60]	CTLA-4, PD-1, PD-L1, CTLA4+PD1	Ipilimumab, Nivolumab, Ipilimumab+Nivolumab, Atezolizumab	Ipilimumab 3mg/kg NR for the rest	Ipilimumab +Nivolumab: Median 4 Atezolizumab: Median 6.5 Ipilimumab: Median 4.5	49	Median 64(32-83)	47/69(68%) male	Melanoma, RCC and Merkel cell carcinoma	IIH	Hypopituitarism	Fatigue, headache, nausea, vomiting	Steroids
Lu et al (2022) [61]	CTLA4, PD1, PDL-1	Ipilimumab, Nivolumab, Pembrolizumab and Cemiplimab, Atezolizumab, Avelumab and Durvalumab	NR	NR	1180	61.9±12.4 (Confirmed PAI) 65.1±10.8(Suspected PAI)	738/1180 (62.5%) male	Melanoma, NSCLC, RCC	NR	Primary adrenal insufficiency	NR	NR
Barnabei et al (2022) [62]	CTLA-4, PD-1, PD-L1, CTLA4+PD1	Ipilimumab, Tremelimumab, Nivolumab, Pembrolizumab, Atezolizumab, Ipilimumab+Nivolumab	NR	NR	11	Median 62	10/11(91%) male	Melanoma, prostate, Bladder, AML, hypopharynx	MRI high-signal intensity of the posterior pituitary may be absent	Anterior + posterior central diabetes insipidus	Weakness, fatigue, headache, visual impairment, anorexia, confusion, memory loss, loss of libido and erectile dysfunction, labile mood, insomnia, temperature intolerance, subjective sensation of fever, and chills	High-dose corticosteroids
Barnabei et al (2022) [63]	CTLA-4, PD-1, PD-L1, CTLA4+PD1, CTLA4+PDL1	Ipilimumab, Nivolumab, Sintilimab, Atezolizumab, Avelumab, Ipilimumab+Nivolumab, Tremelimumab+ Durvalumab	3mg/kg every 21 days in two patients for 4 doses 10mg/kg every 21 days in one patient for 4 doses Tremelimumab 1 mg/Kg 4-weekly for four cycles Durvalumab 20 mg/Kg every four weeks	NR	11	30-74	10/11 Male (90.9%)	Melanoma, Acute myeloid leukemia, Mekel cell carcinoma, Bladder	IIH	Central diabetes insipidus in isolation or as a part of panhypopituitarism	Headache, fatigue, weakness, visual blurriness, and decreased libido, polydipsia and polyuria	Glucocorticoid treatment in all but 2 patients + Vasopressin in 5 patients
Yamada et al (2022) [64]	CTLA-4, PD-1	Ipilimumab, Nivolumab	NR	2-4 cycles	12	65(62-79)	8/12 (67%) Male	RCC (Clear cell /Chromophobe)	IIH	Hypopituitarism	Fatigue, loss of appetite, light-headedness, weight loss, weakness, muscle pain, joint pain	Hydrocortisone at physiological doses.
Ono et al (2022) [65]	CTLA-4, PD-1, PDL-1, CTLA4+PD1	Ipilimumab, Nivolumab, Pembrolizumab, Atezolizumab, Ipilimumab+Nivolumab	NR	2-39 courses	13	A: 71.3±9.1 B: 57.8±11.4	A: 11/13 (84.6%) Male B: 4/4 (50%) Male	NSCLC, malignant melanoma, bladder cancer, RCC and nasal cancer	IIH	Hypopituitarism	Anorexia, fever, fatigue, nausea, diarrhoea, weight loss	NR
Quandt et al (2023) [66]	CTLA4, PD1, PDL1, CTLA-4+PD-1	NR	NR	NR	49	Mean age was 61.3 years	30/49 (61.22%) Male	Melanoma-19/49 (38.8%) and other cancers-30/49 (61.22%)	IIH - enlarged pituitary or empty Sella	Central adrenal insufficiency, abnormal prolactin, Hypogonadotropic hypogonadism	headache, nausea, vomiting, and fatigue, weakness	NR
Cooksley et al (2023) [67]	CTLA-4, PD1, CTLA4+PD1	Ipilimumab, Pembrolizumab, Ipilimumab+Nivolumab	NR	1-13 cycles	14	Median 64 (40–77)	10/14(74.1%) male	Melanoma, RCC, Colorectal, gastric and NSCLC	IIH- normal MRI in 9 patients	Hypopituitarism	Fatigue, headache, nausea and postural dizziness	Oral hydrocortisone (20 mg in the morning, 10 mg at lunchtime and 10 mg in the evening)
Johnson et al (2023) [68]	CTLA-4, PD-1, or PD-L1 or CTLA4+PD1/PDL1	NR	NR	NR	16	Mean 64.9	56.6% Male	Melanoma, RCC, NSCLC, SCLC, NET	IIH	Hypopituitarism	NR	High-dose glucocorticoids
Chiloiro et al (2023) [69]	CTLA-4, PD-1	Ipilimumab, Nivolumab	Ipilimumab: 3mg/kg Nivolumab: 240mg every 15 days	Mean 7.5 cycles	9	Median 59 (SD: 16.8, minimum: 41, maximum: 83)	6/9 (66.66%) male	Melanoma, lung / kidney adenocarcinoma	IIH	Hypopituitarism	Asthenia, diarrhoea, fever	Replacement hydrocortisone
Amylidi et al (2023) [70]	PD-1, CTLA-4+PD-1	Pembrolizumab, Ipilimumab+Nivolumab	NR	NR	4	Median 57	2/4 males	Melanoma, NSCLC, mesothelioma	IIH in 2 patients (Enlargement / enhancement / mild swelling of the pituitary gland)	Hypopituitarism	Case 3 - muscle weakness and fatigue Case 4 - Exhaustion, anorexia, dizziness, and nausea.	Case 3 - only HRT Case 4 - Hydrocortisone
Aviv-Shimoni et al (2023) [71]	CTLA-4, PD-1, PDL-1, CTLA-4+PD-1	Ipilimumab, Nivolumab, Pembrolizumab, Durvalumab, Ipilimumab+Nivolumab, Ipilimumab+Nivolumab+Pembrolizumab	NR	NR	19	56 (IQR 51–69)	7/19 (36.84%) Male	Melanoma, RCC, Kidney, breast, Ovary, Lung	NR	Isolated autoimmune ACTH deficiency	Asthenia, weakness, loss of appetite and/or diarrhoea	Glucocorticoids in a physiologic dose (usually 5 mg prednisone daily)
Galligan et al (2023) [72]	Single-agent ICI(NR), BRAF+MEK inhibitor before ICI	NR	NR	median of 3 cycles (range 1–14)	31	Median age of 60 (IQR 49– 69)	32/51 (62.7%) Male	Melanoma	IIH in 31 patients	Hypopituitarism	Lethargy, headache, anorexia, nausea, visual disturbance, weight loss	Prolonged supraphysiological doses of Glucocorticoids
Hara et al (2023) [73]	CTLA-4, PD-1, PDL-1, CTLA-4+PD-1	Ipilimumab, Pembrolizumab, Nivolumab, Atezolizumab, Durvalumab, Ipilimumab+Nivolumab	NR	Median cycles 8.79 ± 9.64	15	70.0 ± 7.86	10/14 (71.42%) Male	Lung cancer, Malignant melanoma Hodgkin lymphoma, RCC	Magnetic resonance imaging was performed in 11 cases of PD, none of which showed pituitary enlargement.	Hypopituitarism	Fatigue, weight loss, thirst, loss of appetite, and weakness	NR
Fischer et al (2024) [74]	CTLA-4+PD-1, PD-1	Ipilimumab+Nivolumab, Pembrolizumab	NR	NR	28	69.6, 46.2–76.6 (Cases) 66.4, 45.1–79.5 (Controls)	57% male in both groups	Melanoma	(Overall, 10 patients received an MRI of the brain within 24 days prior to diagnosis and 6 days after diagnosis. Among them, 6/10 (60%) had positive findings on MRI). The diagnosis of hypophysitis was made at a median of 83 (range, 26–210) days after the initiation of ICI treatment. FDG PET/CT was acquired at a median of 71 days (range, 5–158) after starting ICI therapy in the case group and a median of 77 days (range, 26–149) in the control group.	Hypopituitarism	Fatigue, weakness, headache, disturbed vision, nausea	NR
Iwama et al (2024) [75]	CTLA4 +PD1	Ipilimumab + Nivolumab	3mg/kg (1mg/kg for NSCLC, RCC mesothelioma)	Ipilimumab: 2.8±1.2(Multi-D) and 2.9±0.9 (IAD) cycles Nivolumab: 3.0 (2.0–6.0) cycles for Multi-D and 4.0 (3.0–7.0) cycles in IAD	41	A - 71 (64–76) B - 68 (63–74)	A - 75% Male (12/16) B- 60% male (15/25)	A - Melanoma >NSCLC, RCC, mesothelioma B - NSCLC > Melanoma >RCC	IIH A - 16 out of 74 (21.6%) B - 25 out of 748 (3.3%)	Hypopituitarism (multi-hormone deficiency)	NR	Brief mention of HRT
Patel et al (2024) [76]	CTLA4, PD1, PDL-1, PD1+CTLA4	NR	NR	Average 37 cycles	37	Mean 65	70% Male	Melanoma, kidney, lung Pleura, Prostate	IIH	Hypopituitarism: HPA axis dysfunction	Fatigue, Nausea, Headache, new-onset confusion, Vomiting, Joint discomfort	Glucocorticoid therapy
Mitri et al (2024) [77]	CTLA4, PD1, CTLA4+PD1	Ipilimumab, Pembrolizumab, Ipilimumab+Nivolumab	Ipilimumab (10 mg/kg; 3 mg/kg) or ipilimumab (3 mg/kg) plus Nivolumab (1 mg/kg) or pembrolizumab (200 mg fixed dose) vs. matched controls	NR	40	Median 59 (20-77)	27/40 (68%) Male	Melanoma	IIH	Hypopituitarism	Weakness, dizziness, altered mental status, abdominal pain, nausea, cephalgia	NR
Van der Leij et al (2024) [78]	CTLA4, PD1, CTLA4+PD1	NR	NR	NR	67	60.4 years (SD 11.3)	63% male	Melanoma, RCC, Lung cancer	IIH	Hypopituitarism	Malaise, fatigue, nausea, anorexia, dizziness, headache, visual symptoms	Low-dose corticosteroids (18/67) Medium-dose corticosteroids (38/67) High-dose corticosteroids (11/67)
Sang et al (2024) [79]	CTLA4, PD1, PDL-1, CTLA4+PD1	Ipilimumab, Nivolumab, Pembrolizumab, Atezolizumab, Avelumab, Durvalumab, Ipilimumab+Nivolumab	Nivolumab: 3mg/kg every 2 weeks Pembrolizumab: 100-200mg every 3 weeks Ipilimumab: 1mg/kg every 3 weeks	Nivolumab: 7 cycles Pembrolizumab: 7-20 cycles Ipilimumab: 3 cycles	13 (no iRAEs)	Mean 59(irH) and 57(non-irH)	irH(69.2% male), non-irH(62.5% male)	Solid organ cancers	IIH - No remarkable finding / normal / poorly enhancing lesion posterior pituitary	Hypopituitarism	Anorexia, nausea, fatigue, dizziness, weight loss, headache	NR
Wang et al (2024) [80]	CTLA-4, PD-1, PD-L1, CTLA4+PD1	Ipilimumab, Nivolumab, Pembrolizumab, Atezolizumab, Durvalumab, Ipilimumab+Nivolumab, Pembrolizumab + Ipilimumab	NR	NR	123	64.3 ± 12.6	79/123 (64.22%) Male	Melanoma and other solid organ tumours	IIH - 88 cases reported normal pituitary gland, 10 cases reported swelling or enlarged pituitary gland or hypophyseal stalk, and 4 cases reported atrophy of the pituitary gland	Hypopituitarism - Isolated ACTH deficiency	Fatigue, nausea, loss of appetite, vomiting, weight loss, diarrhoea, somnolence, malaise	NR
Bando et al (2024) [81]	CTLA-4, PD-1, PDL-1, CTLA4+PD1	Nivolumab, Pembrolizumab,Atezolizumab, Durvalumab, Ipilimumab+Nivolumab	NR	NR	26	68 (62–71)	19/26 (73.08%) Male	Kidney, Lung, Pharynx, Skin, Oesophagus, Stomach, Uterus	Normal MRI	Hypopituitarism	NR	NR
Theiler-Schwetz et al (2024) [82]	CTLA-4+PD-1, PD-1, PDL-1, LAG-3 + PD-1	Ipilimumab+Nivolumab, Nivolumab, Pembrolizumab, Atezolizumab, Relatlimab + Nivolumab	NR	NR	18	Replacement dose group: 64 ±12.6 High dose group: 68.3±5.7	11/18 (61.1%) female (6/10 in replacement group 5/8 in high dose group)	Malignant melanoma, NSCLC, SCC (Skin), RCC, breast cancer, Gastric or Colorectal cancer	IIH in 2 patients. Pituitary microadenomas in 2 cases	Hypopituitarism	Fatigue/ asthenia, loss of appetite, weight loss, hyponatraemia	High-dose glucocorticoid treatment consisted of prednisolone at a dose of 1mg/kg for two weeks followed by taper + HRT
Tang et al (2024) [83]	CTLA4, PD1, PDL1	Nivolumab, Pembrolizumab, Ipilimumab, Atezolizumab	Ipilimumab: 3 or 10mg/kg NR for the rest	NR	1252	45 to 65 (Pembrolizumab: 65 to 80)	NR	Melanoma, NSCLC, Lung, RCC	IIH (Moderately enlarged / enhanced pituitary, thickening of the pituitary stalk)	Hypopituitarism	Headache, fatigue, and weight loss	HRT
Damien Qi et al (2025) [84]	CTLA-4+PD-1, PD-1	Ipilimumab+Nivolumab+Pembrolizumab, Ipilimumab+Nivolumab, Nivolumab, Pembrolizumab	NR	Median treatment duration 11.1 (3.0, 15.0) months	54	64 (54, 70)	38/54 (70%) Male	Melanoma, NSCLC, Gastric, Prostate, Anal SCC, RCC	IIH	Hypopituitarism	Headache, fatigue, GI symptoms	Suggested - Physiological doses of glucocorticoids

**Table 4:** Literature included (but not analysed) for the PDL-1 case reports category Literature included (but not analysed) for the PDL-1 case reports category PD-L1: programmed death-ligand 1; ACTH: adrenocorticotropic hormone; NR: not reported

Author (Year of Publication)	Kanie et al. (2018) [85]	Matthys et al. (2022) [86]	Ogbue et al. (2023) [87]	Furuichi et al. (2023) [88]	Al-Hiari et al. (2024) [89]
Class of ICPi	PD-L1	PD-L1	PD-L1	PD-L1	PD-L1
Name of the drug	Atezolizumab	Durvalumab	Durvalumab	Atezolizumab and Bevacizumab	Atezolizumab and Bevacizumab
Dose/frequency of ICPi	1200 mg every 3 weeks	1500mg	1500 mg every 4 weeks	Not mentioned	Not mentioned
Duration/cycles	19 doses (56 weeks) in case 1, 18 doses (52 weeks) in case 2	2 cycles	5 cycles	8–13 cycles (24–39 weeks)	8 cycles
Total patients	2	1	1	3	1
Gender	Both males	Female	Male	All 3 males	Male
Type of cancer	Non-small cell lung cancer	Neuroendocrine tumour	Non-small cell lung cancer	Hepatocellular carcinoma	Hepatocellular carcinoma
Type of imaging	MRI	MRI	MRI	MRI and CT	MRI
Result of imaging	Hypophysitis	Lymphocytic hypophysitis and limbic encephalitis	Hypophysitis	No abnormality	Hypophysitis
Type of pituitary dysfunction / IRAE	Hypopituitarism	Hypopituitarism	Hypopituitarism (Isolated ACTH deficiency)	Hypopituitarism (secondary adrenal insufficiency)	Hypopituitarism (secondary adrenal insufficiency)
Symptoms	General malaise, appetite loss, diarrhoea, eosinophilia	Memory deficit	Headaches, light-headedness, photophobia, nausea, vomiting, fatigue, night sweats, postural drop, syncope	General fatigue, appetite loss, muscle weakness, weight loss, diarrhoea, eosinophilia, difficulty walking	Generalised weakness, fatigue, confusion, poor oral intake
Acute treatment	Hydrocortisone 15 mg/d	Not mentioned	High-dose prednisone 90 mg/d	Hydrocortisone 15-20 mg/d	Hydrocortisone 100 mg IV loading dose and 50 mg IV doses every 6 hours for 48 hours

Statistical analysis: All statistical analyses were performed using IBM SPSS Statistics version 28.0. Descriptive statistics were employed to summarise the data. Categorical/qualitative variables are presented as frequencies and corresponding percentages. Quantitative variables are expressed as mean ± standard deviation (SD) for normally distributed data, or as median and interquartile range (IQR) for non-normally distributed data. Categorical variables were compared using the Chi-square test or Fisher’s exact test, as appropriate.

Results

A total of 907 articles were identified through the literature search, of which 123 met the eligibility criteria. Ultimately, 84 articles were included in the final review (Figure 1). There were a total of 18 papers, including 1,823 patients who were treated with CTLA-4 agents, out of which 699 were analysed for CTLA-4-associated hypophysitis (Tables 1) [6-23]. Similarly, there were 10 papers including 469 patients who were treated with PD-1/PD-L1 agents, out of which 362 were included in the final analysis for PD-1/PD-L1-associated hypophysitis (Table 2) [24-33]. In comparison, 51 papers included 25,708 patients who were treated with combination therapy involving CTLA-4 and PD-1/PD-L1 agents, out of which 6,198 were included in the final analysis (Table 3) [34-84].

Demographics

In the CTLA-4 group, 69% of participants were male (n = 485) [6-23]. In the PD-1/PD-L1 group, 8 out of 10 studies (n = 327) reported gender-specific data, with males comprising 72% of participants (n = 237) [26-33]. In comparison, the combination therapy group reported 68% male participants (n = 3,283) across 49 of the 51 studies that included gender analysis (n = 4,839) [34-57, 59-82, 84]. Age data were reported with varying completeness and formats across the included studies. In the CTLA-4 group, 17 out of 18 studies (n = 674) reported participant age [6-15, 17-23], with mean ages ranging from 55.5 to 68.2 years and a pooled (unweighted) mean of approximately 63.4 years. Reported age ranges spanned from 31 to 82 years, with standard deviations (SD) ranging from ±1.3 to ±11.2 years, indicating moderate variability. Median ages, where reported, were consistent with the means (60 and 62.1 years). In the PD-1/PD-L1 group, 8 of 10 studies (n = 146) provided age data [26-33], with mean ages ranging from 61.2 to 70.6 years and a pooled unweighted mean of approximately 65.5 years. Age ranges spanned 43 to 80 years, and SDs varied widely (±4.03 to ±15.02). One study using categorical bands reported that 80.4% of participants were younger than 75 years [32]. In the combination therapy group, age was reported in 50 studies (n = 6,176), though formats varied (means, medians, ranges, and age bands) [34-57, 59-84]. Mean ages ranged from 57.7 to 70.0 years, with an unweighted pooled mean of approximately 63.9 years. SDs ranged from ±5.7 to ±14.3 years, and median ages (where reported) ranged from 57 to 65 years. Reported age ranges were broad (15 to 93.4 years), though most participants were between 45 and 80 years.

Dosing

All patients in the CTLA-4 group (n = 699) were treated with ipilimumab [6-23]. Out of 18 papers reviewed, 17 reported dosing information [6-22], with 13 of those specifying exact doses for a total of 268 patients. However, dosing was not specified for 428 patients, and dosing details were not reported (NR) for 3 patients [8,23]. Among the 268 patients with specified doses, 157 received 3 mg/kg, while 111 patients were administered doses greater than 3 mg/kg. Dosing of ipilimumab typically started at 3 mg/kg, with dose escalation to 9 or 10 mg/kg if no objective tumour response was observed after initial doses, provided no grade III/IV toxicity occurred. Several patients received between 3 and 10 mg/kg, with common regimens including 3 mg/kg alone or followed by lower maintenance doses (e.g., 1 mg/kg). Across multiple studies, approximately half of patients were treated with the standard 3 mg/kg dose, while the remainder received escalated doses above 3 mg/kg. Dose escalation protocols and variations were common, with the maximum dose reaching 9 or 10 mg/kg in many cases.

Duration of treatment before development of hypophysitis: 15 out of 18 papers reported the total duration of ipilimumab administration before patients developed hypophysitis (n = 634) [6-16, 19-22]. Ipilimumab is typically given every three weeks for a standard induction of four doses. While most studies followed this schedule, the total number of cycles varied widely, ranging from one to twelve before hypophysitis onset. The average number of cycles usually falls between two and four. Some studies also described maintenance dosing every three months for up to three years after induction. Mean cycles at diagnosis were around 3.3, with total cycles near 4.4, and no significant differences were observed between groups. Overall, patients with hypophysitis showed notable variation in treatment duration, with some receiving as few as two cycles and others up to twelve. Despite this variability, most papers adhered to the every-three-week schedule up to four cycles, with an average of two to four cycles reported before hypophysitis onset.

In the PD-1/PDL1 group (n = 362), the most used PD-1 inhibitors associated with hypophysitis were nivolumab, pembrolizumab, sintilimab, camrelizumab, tislelizumab, and toripalimab. The PD-L1 inhibitors used included avelumab, atezolizumab, and durvalumab.

Dosing

In the PD-1/PD-L1 group, 4 out of 10 studies reported treatment regimens (n = 52), with common regimens including fixed doses or weight-based dosing [26,27,29,30]. Nivolumab was frequently administered at 3 mg/kg every 2 weeks, while pembrolizumab was often given at 2 mg/kg every 3 weeks or as a fixed 200 mg dose every 3 weeks. Camrelizumab and tislelizumab were typically dosed at 200 mg and 240 mg every two weeks, respectively, whereas sintilimab and toripalimab were administered at 200 mg and 240 mg every three weeks, respectively. For PD-L1 inhibitors, atezolizumab was commonly given as a fixed dose of 1200 mg every three weeks. Dosing information for avelumab and durvalumab was NR.

Duration of treatment before development of hypophysitis: Across multiple studies, including data reported in half of the reviewed papers (5 out of 10), the median duration to onset of hypophysitis was approximately 28 weeks after starting PD-1/PDL1 therapy, though this varies widely from 10 to 46 weeks [26,27,29,31,33]. Individual cases demonstrate variability, with symptoms developing after as few as four to six cycles, and others after several months. For example, patients treated with camrelizumab experienced onset between eight and eleven cycles, roughly 16 to 22 weeks [29].

Among a total of 6,198 patients in the combination group, the CTLA-4 inhibitors administered were ipilimumab and tremelimumab; the PD-L1 inhibitors included atezolizumab, avelumab, and durvalumab; and the PD-1 inhibitors used were nivolumab, pembrolizumab, sintilimab, cemiplimab, and spartalizumab.

Dosing

Among combination group therapies reported in 21 out of 51 papers [34,35,37,39,40,42-46,49,58,60,63,69-71,75,77,79,83,84], ipilimumab dosing varied between 1 mg/kg, 3 mg/kg, and 10 mg/kg every 3 to 4 weeks, typically for four cycles, with higher doses associated with an increased risk of hypophysitis. Tremelimumab was typically given at 10 mg/kg every 4 weeks. Nivolumab doses ranged from 1 to 3 mg/kg or a fixed 240 mg every 2 to 3 weeks, often used alone or in combination with ipilimumab. Combination therapy with ipilimumab and nivolumab involves lower ipilimumab doses (1-3 mg/kg) alongside nivolumab, administered over 3 to 4 cycles. Pembrolizumab was administered at 2 mg/kg or 100-200 mg every 3 weeks. Additionally, atezolizumab was dosed at 1200 mg every 3 weeks, and durvalumab at 20 mg/kg every 4 weeks. These regimens reflect the diversity of dosing strategies used in combination ICPi treatments across studies. Treatment durations and cumulative doses vary widely, reflecting different protocols and patient populations. Overall, the dosing regimens highlight a balance between efficacy and immune-related adverse event risks, particularly with higher ipilimumab doses.

Duration of treatment before development of hypophysitis: Out of 51 reviewed studies, 22 reported treatment duration data for immune checkpoint inhibitors (n = 1273) [34,35,37,39,40,42-46,49,58,60,63,69-71,75,77,79,83,84]. Median treatment cycles before they developed hypophysitis varied by drug: nivolumab was administered for approximately 7 cycles, pembrolizumab ranged from 7 to 20 cycles, and ipilimumab was typically given for 3 to 4.5 cycles. Combination therapy with ipilimumab plus nivolumab showed a median of 4 cycles before hypophysitis onset. For anti-PD-1 agents overall, the median treatment duration was about 6.5 cycles. These data suggest that hypophysitis often occurs within the first several cycles, particularly with ipilimumab-containing regimens, which tend to have shorter treatment durations compared to anti-PD-1 monotherapies. This highlights the importance of close monitoring during early treatment cycles to manage potential toxicities effectively.

Imaging

In the CTLA-4 group, all 699 patients underwent MRI [6 to 23], out of which 235 had radiological evidence of hypophysitis. The most common biochemical abnormality in the CTLA-4 group was hypopituitarism. Similarly, in the PD-1/PD-L1 group, imaging modalities were reported in 8 out of 10 studies (n = 101) [25,26-31,33]. MRI was used in all reported cases, with 53 patients showing radiological evidence of hypophysitis. Additionally, 13 patients exhibited nonspecific abnormalities such as stalk abnormalities or empty sella, while 35 patients had normal scans. The most common biochemical abnormality in the PD-1/PD-L1 group was isolated ACTH deficiency. In the combination group, imaging modalities were reported in 46 out of 51 studies [34,36-54,56,57,60,62-70,72-84], comprising a total of 4,685 patients. Among these, 44 studies used MRI, one study used biopsy [35], and one study used FDG-PET/CT in combination with either CT or MRI [72]. Reported abnormalities included radiological evidence of hypophysitis, pituitary stalk abnormalities, pituitary enlargement, microadenoma, pituitary atrophy, and empty sella. Some patients showed no radiological abnormalities. The most common biochemical abnormalities in the combination group were hypopituitarism and secondary AI. Overall, while MRI is a key diagnostic tool, it may not always detect subtle or early-stage pituitary involvement, underscoring the importance of integrating clinical, biochemical, and radiological data in the evaluation of ICI-related pituitary dysfunction. Treatment: In CTLA-4-associated hypophysitis, reported in 17 out of 18 studies [6-10, 12-23], high-dose glucocorticoids such as dexamethasone or prednisolone at 1 mg/kg for approximately two weeks are commonly administered, followed by a tapering regimen to reach a physiological maintenance dose. Hormone replacement therapy (HRT) is often necessary, including hydrocortisone or low-dose prednisolone, fludrocortisone, thyroxine, desmopressin (DDAVP), and sex hormone replacements, depending on the specific hormone deficits. Isolated ACTH deficiency (IAD), a frequent outcome, is generally irreversible and requires lifelong corticosteroid replacement. In PD-1/PD-L1 associated hypophysitis, reported in 7 out of 10 studies [25-27,29-32], treatment typically involves intravenous corticosteroids with a median dose of 200 mg (range: 50-300 mg), followed by maintenance therapy using low-dose prednisolone or hydrocortisone (2.5-7.5 mg/day). For combination therapy (CTLA-4 plus PD-1/PD-L1), 35 out of 51 studies [34,35,37-40,42,43,46,47,49,50,53-56,59,60,62-64,67-72,75,76,78,80-84] reported similar use of high-dose supraphysiological corticosteroids with a taper to physiological doses, along with comprehensive HRT as needed. Across all ICI types, high-dose glucocorticoid initiation, careful tapering, and tailored long-term hormone replacement remain the mainstays of management.

Discussion

The advent of immune checkpoint inhibitors (ICPis) has significantly transformed the therapeutic landscape of oncology, with an ever-growing number of indications across a wide spectrum of malignancies. However, immune-related adverse events (irAEs) remain a substantial clinical concern, among which endocrinopathies are particularly common [90,91]. Hypophysitis is a notable endocrine irAE linked to ICPi therapy, presenting diagnostic and management challenges due to its nonspecific symptomatology, frequent concomitant use of exogenous glucocorticoids, and the potential for long-term or permanent multi-hormonal deficiencies resulting from panhypopituitarism [92].

Demographically, the weighted average percentage of male participants across all three treatment groups was approximately 68.3%. Age distributions were similar, with pooled mean ages in the early to mid-60s, though reporting heterogeneity was noted.

All patients in the CTLA-4 inhibitor group received ipilimumab, with 3 mg/kg being the most commonly used dose in clinical practice. Higher doses were reserved for escalation in selected responsive and tolerable cases. Hypophysitis associated with ipilimumab, or CTLA-4 combination therapy, tended to be more severe and occurred earlier compared to that associated with PD-1/PD-L1 inhibitors. Our analysis confirms that ICPi-induced hypophysitis typically emerges within the first few months of therapy but may present at variable time points depending on treatment modality and patient-specific factors.

In terms of cancer types treated, the CTLA-4 inhibitor group primarily included melanoma, renal cell carcinoma, and prostate cancer (Table 1). The PD-1/PD-L1 group covered a broader range of malignancies, including metastatic melanoma, non-small cell lung cancer (NSCLC), small cell lung cancer (SCLC), renal cell carcinoma, bladder cancer, hepatocellular carcinoma (HCC), oesophageal cancer, colorectal cancer, gastric cancer, nasopharyngeal carcinoma, intrahepatic cholangiocarcinoma, breast cancer, cervical cancer, ovarian cancer, cutaneous T-cell lymphoma, and head and neck squamous cell carcinoma (HNSCC)(Table 2). The combination therapy group included the full spectrum of cancers treated with PD-1/PD-L1 agents, in addition to malignancies such as pleural mesothelioma, acute myeloid leukaemia (AML), chronic myeloid leukaemia (CML), Hodgkin lymphoma, papillary thyroid carcinoma, and cutaneous squamous cell carcinoma (cSCC) (Table 3). This distribution reflects the expanding use of ICPis across both solid tumours and haematologic malignancies, with combination therapy employed in the most diverse range of indications.

Clinical presentations showed overlapping but distinct symptom profiles across treatment groups. In the CTLA-4 group, commonly reported symptoms included headache, fatigue, visual disturbances, dizziness, hyponatraemia, decreased libido, nausea or anorexia, confusion, mood changes, weight loss, hot flushes, and myalgias/arthralgias (Table 1). The PD-1/PD-L1 group presented with fatigue, headaches, nausea or vomiting, anorexia, hyponatraemia, hypotension, and myalgias/arthralgias. Some reports also described dizziness, altered mental status, visual changes, fever, amenorrhoea, hypoglycaemia, aspergillosis, and memory loss (Table 2). The combination therapy group exhibited the broadest symptom spectrum, including fatigue, confusion, hypotension, headache, visual disturbances, hyponatraemia, anorexia, weight loss, nausea, vomiting, diarrhoea, somnolence, dizziness, altered mental status, eosinophilia, fever, rash, syncope, psychiatric symptoms (e.g., apathy, anxiety, memory loss, labile mood, lethargy), insomnia, loss of libido, erectile dysfunction, and myalgias/arthralgias (Table 3).

Our systematic review highlights hypopituitarism as a frequent and often persistent consequence of ICPi-associated hypophysitis, emphasising the importance of early recognition to mitigate long-term endocrine morbidity. Hypophysitis induced by CTLA-4 inhibitors, particularly ipilimumab and CTLA-4-based combination therapies, is more commonly associated with hypopituitarism than that caused by other ICPi classes. In contrast, isolated adrenocorticotropic hormone (ACTH) deficiency, more frequently observed with PD-1 and PD-L1 inhibitors, typically presents as secondary AI, often without distinct abnormalities on early MRI imaging. High-dose glucocorticoid initiation, careful tapering, and tailored long-term hormone replacement remained the mainstays of management.

Limitations

Several limitations should be acknowledged in this review. First, significant heterogeneity existed among the included studies in terms of study design, patient populations, cancer types, dosing regimens, and follow-up durations, which may limit the generalizability of the findings. Reporting inconsistencies, particularly regarding dosing details, duration of therapy, and timing of hypophysitis onset, constrained the ability to perform quantitative meta-analysis and may introduce bias.

Age and gender data were variably reported, and some studies used categorical age bands rather than continuous measures, complicating pooled demographic analyses. Imaging modalities and criteria for radiological diagnosis of hypophysitis also varied, potentially affecting the sensitivity and specificity of detection across cohorts.

Moreover, most data were derived from retrospective observational studies and case series, which are subject to selection bias, incomplete data capture, and variable diagnostic criteria. The lack of standardised protocols for the diagnosis and management of ICI-related hypophysitis across studies further complicates direct comparisons and synthesis of therapeutic outcomes.

Finally, long-term follow-up data on endocrine recovery and patient outcomes post-hypophysitis were limited, impeding a comprehensive understanding of prognosis and optimal hormone replacement strategies. Future prospective multicentre studies employing standardised diagnostic criteria, uniform treatment protocols, and systematic long-term monitoring are essential to enhance understanding of the pathophysiology, clinical course, and optimal management of ICI-associated hypophysitis.

Future scope/research

The future scope of ICPi-associated hypophysitis involves several key areas to enhance diagnosis, management, and outcomes. Prospective, multicentre studies with standardised diagnostic criteria-encompassing clinical, biochemical, and radiological parameters-are essential to ensure consistency and improve generalisability across diverse populations and cancer types. Longitudinal follow-up is crucial to better understand the natural history, rates of endocrine recovery, and long-term outcomes such as quality of life and survival. Further research into predictive biomarkers and underlying mechanisms will support early detection and risk stratification. Comparative studies examining different immune checkpoint inhibitor dosing regimens and treatment durations are needed to optimise oncologic efficacy while minimising endocrine toxicity. Finally, the development and validation of standardised management protocols, including glucocorticoid therapy and hormone replacement strategies, will be critical for improving the care and outcomes of affected patients.

## Conclusions

This systematic review delineates the distinct clinical, radiological, and therapeutic profiles of immune checkpoint inhibitor (ICPi)-associated hypophysitis across CTLA-4 inhibitor monotherapy, PD-1/PD-L1 inhibitor monotherapy, and combination ICPi regimens.CTLA-4 inhibitors, particularly ipilimumab, are linked to earlier onset and multifaceted hypopituitarism with more frequent MRI abnormalities, while PD-1/PD-L1 inhibitors typically cause delayed, isolated ACTH deficiency with variable imaging findings. Combination therapy presents the widest clinical variability and greatest risk of hypophysitis. High-dose glucocorticoid therapy remains the primary intervention, with subsequent tapering to physiological maintenance doses complemented by individualised HRT. Notably, MRI sensitivity is limited for early or subtle pituitary involvement, necessitating a multidisciplinary diagnostic approach integrating clinical assessment and endocrine evaluation. Vigilant monitoring and early, multidisciplinary diagnosis are essential to reduce morbidity and optimise outcomes in patients receiving immune checkpoint therapy.
